# Supplementary material for: Patterns of Intron Gain and Loss in Fungi
Source: PLoS Biol. 2004 Nov 30;2(12):e422. doi: 10.1371/journal.pbio.0020422 (PMC532390; doi:10.1371/journal.pbio.0020422)
Supplement: Table S1 — Also available at http://genes.mit.edu/NielsenEtAl/. (4.3 MB ZIP). [file pbio.0020422.st001.zip › NielsenEtAl/html/208.html]

AN7725.1.NCU06550.1.MG05980.1.FG05035.1


```
 CLUSTAL W (1.82) Multiple Sequence Alignments - Introns Inserted


Sequence 1: MG05980.1	319 aa
Sequence 2: FG05035.1	311 aa
Sequence 3: AN7725.1	304 aa
Sequence 4: NCU06550.1	308 aa
Alignment Length: 325 aa
Number Identitical Residues: 187 aa
Alignment Score (without introns) 8161


MG05980.1 	MASTTSNGNGHPASATNGSNGTSNIPSFAVKAGLAQMLKGGVIMDVTNAEQ~ARIAEEAG
NCU06550.1	MTSTST--------TTNGDS-------FTVKAGLAQMLKGGVIMDVTTPAE~ARIAEEAG
FG05035.1 	MTNDNSNNTG----ASNGEAKSS----FTVKAGLAQMLKGGVIMDVVNAEQ~ARIAEEAG
AN7725.1  	MAATNG--------ASND---------FTVKAGLAQMLKGGVIMDVVNAEQ0ARIAEEAG
          	*:  .         ::*.         *:*****************... : ********

MG05980.1 	ACAVMALERVPADIRRDGGVARMSDPAMIREIKAAVTIPVMAKARIGHFVEAQ~ILESLE
NCU06550.1	ACAVMALERIPSDIRAAGGVARMSNPSMIKEIQAAVTIPVMAKARIGHVTECR~ILEQLG
FG05035.1 	ACAVMALERVPADIRKDGGVARMSDPAMIKEIQDAVTIPVMAKARIGHFVECQ~ILEALG
AN7725.1  	AAAVMALERVPADIRAQGGVARMSDPSMIKEIMEAVTIPVMAKARIGHFVECQ0ILEAIG
          	*.*******:*:***  *******:*:**:**  **************..*.: *** : 

MG05980.1 	VDYVDESEVLTPADEKYHIEKSDFAVPFVCGCRNLGEALRRIAEGAAMIRTKGEAGTGDV
NCU06550.1	VDYIDESEVLTPADDTYHVQKDQFKAPFVCGCRNLGEALRRIKEGAAMIRTKGEAGTGDV
FG05035.1 	VDYIDESEVLTPADDESHVEKSPFSVPFVCGCRNLGEALRRIAEGAAMIRTKGEAGTGDV
AN7725.1  	VDYIDESEVLTPADNLYHVTKHNFKAPFVCGCRNLGEALRRISEGAAMIRTKGEAGTGDV
          	***:**********:  *: *  * .**************** *****************

MG05980.1 	VQAVTHMRTLKADIARAKAALNEGG--MPALRAIARELECDPALLKQTVDLGRLPVVNFA
NCU06550.1	VEAVKHIRTVNAEIAQAKAALASGG--ELAVAKMAREIGADVELLKKTAELGRLPVVNFA
FG05035.1 	VEAVRHMKTVNRDIAQAKAALAEGG--IVRIREMARKLEVDAELLRQTAELGRLPVVNFA
AN7725.1  	VEAVKHMRTVNAEIARARAILQSSPDPEPELRAYARELEAPYELLREAAEKGRLPVVNFA
          	*:** *::*:: :**:*:* * .. ..   :   **::     **:::.: *********

MG05980.1 	AGGIATPADAALMMQLGCDGVFVGSGIFKSGD----PAKRAKAIVQATTHFRDAKMLAEY
NCU06550.1	AGGVATPADAALMMEFGCDGVFVGSGIFKDAKTPEHALQRARAVVKAVANWNDYGALVEA
FG05035.1 	AGGVATPADAALMMQLGCDGVFVGSGIFKSGD----PAKRAKAIVRATTHFRDAKVLAET
AN7725.1  	AGGVATPADAALMMQLGCDGVFVGSGIFKSGD----AKKRAKAIVQAVTHYKDPKVLAEV
          	***:**********::*************...    . :**:*:*:*.:::.*   *.* 

MG05980.1 	SSGLGEAMVGINCDSMQQSEKLAVRGW
NCU06550.1	CIEHGEAMKGISNAGMKPEERMAGRGW
FG05035.1 	STGLGEAMVGINCDSMKPEEKLAGRGW
AN7725.1  	SQGLGEAMVGINVSHMKDEDKLAKRGW
          	.   **** **.   *: .:::* ***
```
